# The Induction of Tumours with Nitrogen Mustards

**DOI:** 10.1038/bjc.1949.14

**Published:** 1949-03

**Authors:** E. Boyland, E. S. Horning

## Abstract

**Images:**


### THE INDUCTION OF TUMOURS WITH NITROGEN MUSTARDS.

E. BOYLAND AND E. S. HORNING.
*From the Chester Beatty Research Institute of the Royal Cancer Hospital, London, S.W.3.*

Received for publication December 12, 1948.

RECENT experience has shown that many of the growth-inhibiting agents used in the palliative treatment of cancer are carcinogenic. They also cause specific damage to cell nuclei and chromosomes and are able to induce mutations. The association of these biological effects of (1) growth inhibition, (2) chromosome damage, (3) production of mutations and (4) induction of cancer suggests that they may have a common fundamental biochemical mechanism. If the induction of cancer is indeed a somatic mutation, then cancer induction might be included as a special mutation. The fact that X-rays can produce cancer in man was published in 1902 (Frieben, 1902), seven years after Röntgen's discovery of X-rays. Müller (1928) found that X-rays are mutagenic, and later Mather and Stone (1933) and Koller (1934) described the chromosome damage following irradiation. The idea that cancer arises as a somatic mutation was supported by the demonstration of an increased incidence of mutations occurring in mice treated with chemical carcinogens. This was shown with methylcholanthrene by Strong (1945) and with 1:2:5:6-dibenzanthracene by Carr (1947). The carcinogenic hydrocarbons inhibit the growth of animals and the establishment and growth of transplanted tumours (Haddow, Scott and Scott, 1937). They are able to cause breaking of chromosomes in tumour cells (Koller, personal communication). One of the most potent carcinogenic hydrocarbons, 9:10-dimethyl-1:2-benzanthracene, has been used with some temporary success in the treatment of lymphocytic leukaemia (Engelbreth-Holm and Stamer, 1947).

In the treatment of leukaemia (Paterson, ApThomas, Haddow and Watkinson, 1946), urethane has been used as an alternative to radio-therapy. This drug also induces cancer of the lung in mice (Nettleship and Henshaw, 1943) and inhibits mitosis (Dustin, 1947). Derivatives of 4-dimethylaminostilbene also have this dual action. These compounds were first found to be growth inhibitors, and later found to be potent carcinogens (Haddow, Harris, Kon and Roe, 1948).

The vesicants, mustard gas (di(2-chloroethyl)sulphide) and nitrogen mustards (e.g. methyl di(2-chloroethyl)amine, HN2 and tri(2-chloroethyl)amine HN3) cause increased stickiness of chromosomes and rupture of chromosomes (Darlington and Koller, 1947; Boyland, Clegg, Koller, Rhoden and Warwick, 1948). They also produce mutations, as was shown in drosophila by Auerbach and Robson (1947) and in neurospora by Horowitz, Houlahan, Hungate and Wright (1946) and by Tatum (1947). The numerous other radiomimetic actions of the nitrogen mustards, which have been discussed elsewhere (Boyland, 1948), show the remarkable similarity in action of vesicants and radiations. Mustard gas was shown to have an anticarcinogenic action by Berenblum (1931), and this antagonistic action may be similar to the antagonism of weak carcinogens to potent carcinogens described by Lacassagne, Buu-Hoï and Cagniant (1944). By analogy with previous findings the nitrogen mustards might be expected to have carcinogenic action, and the actual induction of tumours by these substances is described in the present paper.

### EXPERIMENTAL.

Two groups each of 20 stock mice were given weekly subcutaneous injections of 10 ml. per kg. body-weight of freshly made aqueous solutions of nitrogen mustard. The first group was given methyl di(2-chloroethyl)amine hydrochloride (HN2) in a concentration of 1 mg. per 10 ml., so that it received 1·0 mg. per kg. body-weight at weekly intervals for 50 weeks. The second group received the same dose of tri(2-chloroethyl)amine hydrochloride (HN3) for 10 weeks, after which time only 4 mice remained alive and injections were stopped.

Post-mortem findings of the mice which died within a week of an injection usually showed congestion of the alimentary tract or lungs. With the doses used no nervous symptoms were seen. All the coloured mice showed the effect of greying of hair (Boyland *et al.*, 1948) similar to that produced by X-rays (Hance and Murphy, 1926) or by subcutaneously implanted plutonium (Prosser, Painter, Lisco, Brues, Jacobson and Swift, 1947). The de-pigmented area of fur usually became visible at the site of injection after 2 or 3 weeks. Other experiments have shown that intradermal injection of the nitrogen mustard is more effective than subcutaneous injection in producing this effect on hair colour.

### *Delayed effects of treatment.*

Differential blood counts were made on the surviving mice 15 months after the beginning of the injections. Three of the HN2 series (No. 16, 18 and 19) and three of the HN3 series (No. 38, 39 and 40) were leucopenic. The blood picture was abnormal (Table I) in showing a deficiency of lymphocytes. Cameron, Courtice and Jones (1947) found that injection of HN2 into rabbits and dogs caused neutropenia and lymphocytopenia. The present results indicate that the repeated injection of nitrogen mustards induces a chronic lymphocytopenia in a proportion of treated mice.

One mouse (No. 10) which died 148 days after beginning of treatment with HN2 showed (1) slight proliferation of the bronchiolar epithelium, (2) mobilization of lymphocytes round ducts and blood vessels of the liver, and (3) aggregation of lymphocytes around bronchioles and blood vessels of the lung.

TABLE I.—*Blood Counts of Mice made* 13 *Months after Commencement of Treatment.*

Mice No. 14–20 treated with HN2.
Mice No. 37–40 treated with HN3.

| Mouse No. | R.B.C. count in millions. | Hb % (Haldane). | Total leucocytes. | Differential count of per cent of total. Neutrophil polymorphs. | Lymphocytes. | Monocytes. | Eosinophils. | Unclassified immature cells. |
|---|---|---|---|---|---|---|---|---|
| 14 | 6·4 | 44 | 46,400 | 66 | 31 | 1 | .. | 2 |
| 15 | 7·3 | 72 | 46,400 | 70 | 24 | 1 | .. | 5 |
| 16 | 3·3 | 38 | 10,000 | 30 | 66 | .. | .. | 4 |
| 17 | 7·3 | 73 | 8,000 | 35 | 62 | 3 | .. | .. |
| 18 | 9·2 | 88 | 16,000 | 45 | 50 | 5 | .. | .. |
| 19 | 10·1 | 96 | 12,600 | 44 | 52 | 3 | 1 | .. |
| 20 | 10·6 | 103 | 14,600 | 60 | 38 | 2 | .. | .. |
| 37 | 10·1 | 110 | 18,400 | 41 | 53 | 1 | .. | 5 |
| 38 | 11·9 | 110 | 9,400 | 58 | 41 | .. | .. | 1 |
| 39 | 10·5 | 108 | 6,000 | 32 | 67 | 1 | .. | .. |
| 40 | 9·8 | 94 | 5,600 | 39 | 69 | 2 | .. | .. |
| Untreated (mean of 20 mixed stock mice) | 10·2 | 103 | 19,000 | 17 | 79 | 2 | 1 | 1 |

The post-mortem findings in mice which survived more than 280 days are shown in Tables II and III. Of the total of 14 mice, 10 had tumours, including lung carcinomas and adenomas in 8, lymphosarcomas in 2, a uterine fibromyoma and a sarcoma at the site of injection. Of the mice free of neoplasms 2 had lymphocytic infiltration of the liver and lung and 2 had abnormal proliferation of the bronchiolar epithelium. Most of the mice with lung tumours or lymphosarcomas in the liver had enlarged lymph nodes.

A group of 40 untreated mice from the same source as the experimental mice were killed when between 14 and 18 months of age. In this series 6 mice had adenomas of the lung, 2 hepatomas and 3 had enlarged lymph nodes.

The mice with lymphosarcomas had greatly enlarged livers (Fig. 1), in which the normal structure was destroyed (Fig. 2), the malignant cells showing numerous mitoses (Fig. 3).

The lung tumours were for the most part larger and more malignant (Fig. 4 and 5) than those occurring spontaneously in mice. Many of these lung tumours

---

DESCRIPTION OF PLATES.

FIG. 1.—Mouse 12 (decapitated). Treated for 347 days with HN2. This rodent developed a lymphosarcoma in the liver, together with enlarged thymus and lymph nodes. Macroscopic lesions can be seen in liver. × 1¼.

FIG. 2.—Lymphosarcoma; section of liver of mouse seen in Fig. 1. × 105.

FIG. 3.—Mouse 40. Subcutaneous spindle-celled sarcoma which developed at site of injection with HN3. × 210.

FIG. 4.—Mouse 15. Showing dense aggregation of lymphocytes around bronchioles in lung following treatment with HN2. × 58.

FIG. 5.—Mouse 17. Adenocarcinoma of lung following treatment with HN2. × 105.

FIG. 6.—Mouse 38. Carcinoma of lung following treatment with HN3. Tumour cells are seen invading the alveolar tissue of the lung. × 210.

FIG. 7.—Same as Fig. 6. Showing another field in the same section, in which the lumen of a bronchiole is occluded by tumour cells. × 210.

FIG. 8.—Mouse 19. Treated with HN2. Carcinoma of lung. × 210.

Boyland and Horning.

5

6

7

8

TABLE II.—*Mice Injected with Weekly Doses of* 1·0 *mg./kg. of Methyl Di*-(2-*chloroethyl*)*amine Hydrochloride* (*HN*2) *till Death or for* 50 *Weeks.*

| Mouse No. | Survival time (days). | Post-mortem findings. |
|---|---|---|
| 10 | 148 | Proliferation of bronchiolar epithelium with aggregation of lymphocytes round ducts and vessels of liver. |
| 11 | 284 | *Lung*, adenoma. |
| 12 | 347 | *Liver*, lymphosarcoma with dense lymphocytic infiltration and cell degeneration. *Lymph nodes* and *thymus* greatly enlarged. |
| 13 | 367 | *Lung*, proliferation of bronchioles; early bronchogenic tumours and thickening of interalveolar septa. |
| 14 | 398 | *Liver*, slight lymphocytic infiltration.<br>*Lung*, abscess, destruction of alveoli, dense lymphocytic infiltration with polymorphs. |
| 15 | 399 | *Liver*, marked lymphocytic infiltration.<br>*Lung*, thickening of interalveolar septa. |
| 16 | 550 | *Uterus* fibromyoma.<br>*Lung*, proliferation of bronchiolar epithelium with fibrosis and internal infiltration of lymphocytes in blood vessel. |
| 17 | 569 | *Lung*, carcinoma accompanied by dense lymphocytic infiltration.<br>*Liver*, slight lymphocytic infiltration accompanied by degeneration of liver cells.<br>*Spleen*, *thymus* and *lymph nodes* enlarged. |
| 18 | 569 | *Lung*, *carcinoma* with aggregation of lymphocytes around blood vessels and bronchioles.<br>*Prostate*, hypertrophy of prostatic epithelium, not so pronounced as in No. 40.<br>*Lymph nodes* enlarged. |
| 19 | 575 | *Lung*, carcinoma. |
| 20 | 580 | *Liver*, marked lymphocytic infiltration around blood vessels and ducts. |

TABLE III.—*Mice Injected with* 10 *Doses of* 1·0 *mg./kg. of Tri*-(2-*chloroethyl*)*amine Hydrochloride* (*HN*3) *which Survived More than* 54 *Days.*

| Mouse No. | Survival time (days). | Post-mortem findings. |
|---|---|---|
| 37 | 548 | *Lung* adenoma with lymphocytic infiltration. |
| 38 | 552 | *Lung*, bronchogenic carcinoma infiltrating alveolar tissue. |
| 39 | 560 | *Lung*, bronchiolar proliferation.<br>*Kidney*, glomerulonephritis. |
| 40 | 567 | *Lung*, carcinoma.<br>Spindle-celled *sarcoma* at site of injection.<br>*Prostate*, pronounced hypertrophy of epithelium in anterior lobe.<br>*Liver*, lymphocytes around duct.<br>*Thymus*, *lymph nodes* and *parotid* enlarged. |

were invading the normal tissue, and in many cases the lungs of these mice had aggregations of lymphocytes.

The spindle-celled sarcoma at the site of injection of mouse No. 40 was still small when the mouse was killed, and was immediately below the area of depigmented skin where injections had been made.

### DISCUSSION.

The results of the experiments suggest that the nitrogen mustards have a slow carcinogenic action. As only 14 mice survived to an age when tumours occur the experiments are being extended in mice of pure strains. The incidence of lung tumours and lymphosarcomas was higher than would be expected to occur spontaneously, and the tumours were larger and more malignant than those generally encountered in untreated mice.

The carcinogenic action of the nitrogen mustards is very slow, the earliest lung tumour and lymphosarcoma being seen at 284 and 347 days respectively. Berenblum and Shubik (1947) have shown that carcinogenesis can be divided into an initial stage in which cells are irreversibly changed into latent tumour cells, and a development stage which can be brought about by cocarcinogens such as croton oil. The incidence of tumours expressed as quantal response is probably dependent on the initiating action, but the rate of appearance of the tumours depends upon cocarcinogenic action.

The present authors consider that the initial irreversible conversion to latent tumour cells is the change which is associated with chromosome damage and mutations. The nitrogen mustards are possibly active in initiating the carcinogenic change but poor in developing action. In order to test this hypothesis experiments are in hand in which mice are being treated with nitrogen mustard and croton oil.

A possible explanation of the association between inhibition of growth and carcinogenic power is that the inhibition is due to interference with cell division in growing tissues. The drugs under discussion reduce the rate of growth by impeding mitosis. This interference with mitosis is likely to increase the chances of a somatic mutation from normal to malignant cells occurring. The somatic mutation is possibly a relatively rare event, the occurrence of which is more probable when the difficulties of cell division are increased either by radiations or by chemical agents.

### SUMMARY.

Mice were given weekly subcutaneous injections of nitrogen mustards. Of 14 mice which survived more than 250 days, 10 had tumours including lung tumours (8), lymphosarcomas (2), a uterine fibromyoma and a spindle-celled sarcoma at site of injection.

This investigation has been supported by grants to The Royal Cancer Hospital from the British Empire Cancer Campaign, the Jane Coffin Childs Memorial Fund for Medical Research, the Anna Fuller Fund, and the U.S. Public Health Service.

### REFERENCES.

AUERBACH, C., AND ROBSON, J. M.—(1947) *Proc. Roy. Soc. Edinb.*, B, **62**, 284.

BERENBLUM, I.—(1931) *J. Path. Bact.*, **34**, 731.

*Idem* AND SHUBIK, P.—(1947) *Brit. J. Cancer*, **1**, 383.

BOYLAND, E.—(1948) *Biochem. Soc. Symposia*, **2**, 61.

*Idem*, CLEGG, J. W., KOLLER, P. C., RHODEN, E., AND WARWICK, O. H.—(1948) *Brit. J. Cancer*, **2**, 17.

CAMERON, G. R., COURTICE, F. C., AND JONES, R. P.—(1947) *J. Path. Bact.*, **59**, 425.

CARR, J. G.—(1947) *Brit. J. Cancer*, **1**, 152.

DARLINGTON, C. D., AND KOLLER, P. C.—(1947) *Heredity*, **1**, 187.

DUSTIN, P.—(1947) *Brit. J. Cancer*, **1**, 48.

ENGELBRETH-HOLM, J., AND STAMER, S.—(1947) 'Approaches to Tumour Chemotherapy.' Washington, D.C. (Amer. Ass. Adv. Sci.), p. 419.

FRIEBEN, A.—(1902) *Fortschr. Röntgenstr.*, **6**, 106.

HADDOW, A., HARRIS, R. J. C., KON, G. A. R., AND ROE, E. M. F.—(1948) *Phil. Trans.*, A, **241**, 147.

*Idem*, SCOTT, C. M., AND SCOTT, J. D.—(1937) *Proc. Roy. Soc.*, B, **122**, 477.

HANCE, R. T., AND MURPHY, J. B.—(1926) *J. exp. Med.*, **44**, 339.

HOROWITZ, N. H., HOULAHAN, M. B., HUNGATE, M. G., AND WRIGHT, B.—(1946) *Science*, **104**, 233.

KOLLER, P. C.—(1934) *Genetics*, **16**, 447.

LACASSAGNE, A., BUU-HOÏ, AND CAGNIANT, P.—(1944) *C.R. Soc. Biol. Paris*, **138**, 16.

MATHER, K., AND STONE, L. M. A.—(1933) *J. Genet.*, **28**, 1.

MULLER, H. J.—(1928) *Proc. nat. Acad. Sci. Wash.*, **14**, 714.

NETTLESHIP, A., AND HENSHAW, P. S.—(1943) *J. nat. Cancer Inst.*, **4**, 309.

PATERSON, E., HADDOW, A., APTHOMAS, I., AND WATKINSON, J. M.—(1946) *Lancet*, i, 677.

PROSSER, C. L., PAINTER, E. E., LISCO, H., BRUES, A. M., JACOBSON, L. O., AND SWIFT, M. N.—(1947) *Radiology*, **49**, 299.

STRONG, L. C.—(1945) *Proc. nat. Acad. Sci. Wash.*, **31**, 290.

TATUM, E. L.—(1947) *Ann. N.Y. Acad. Sci.*, **49**, 97.

---


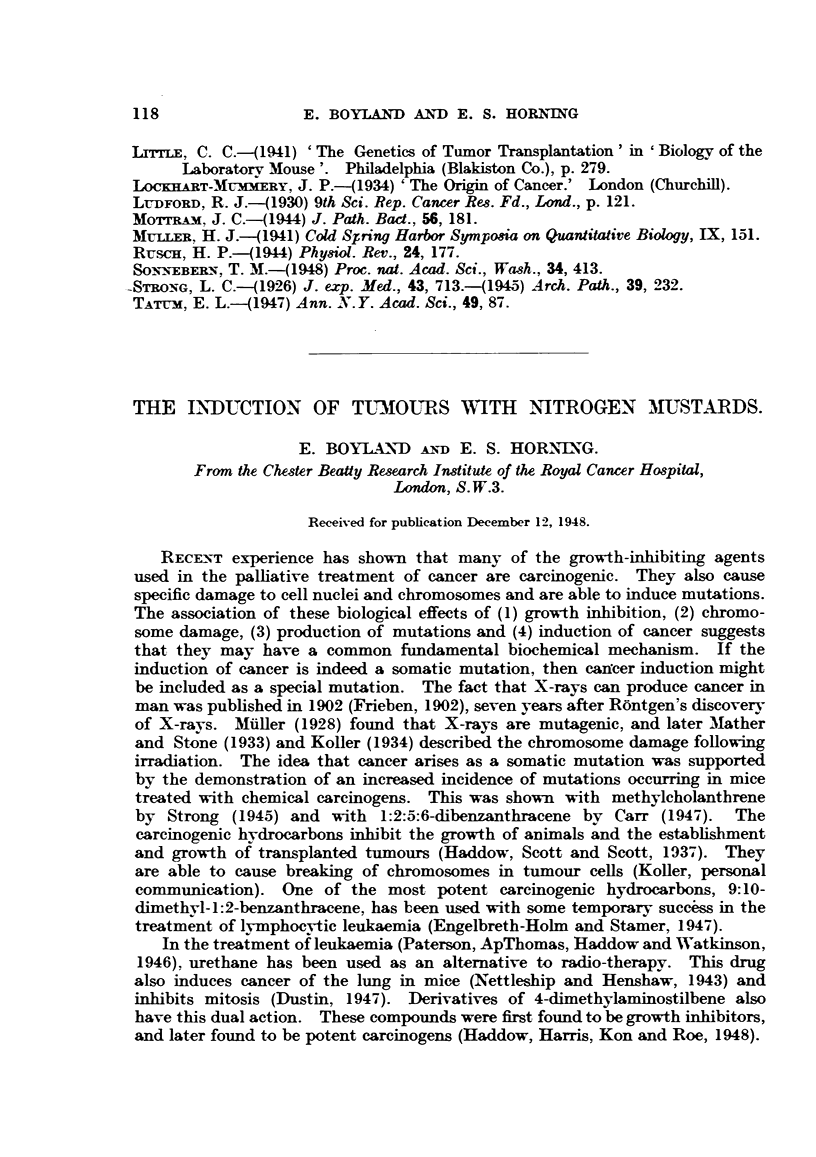

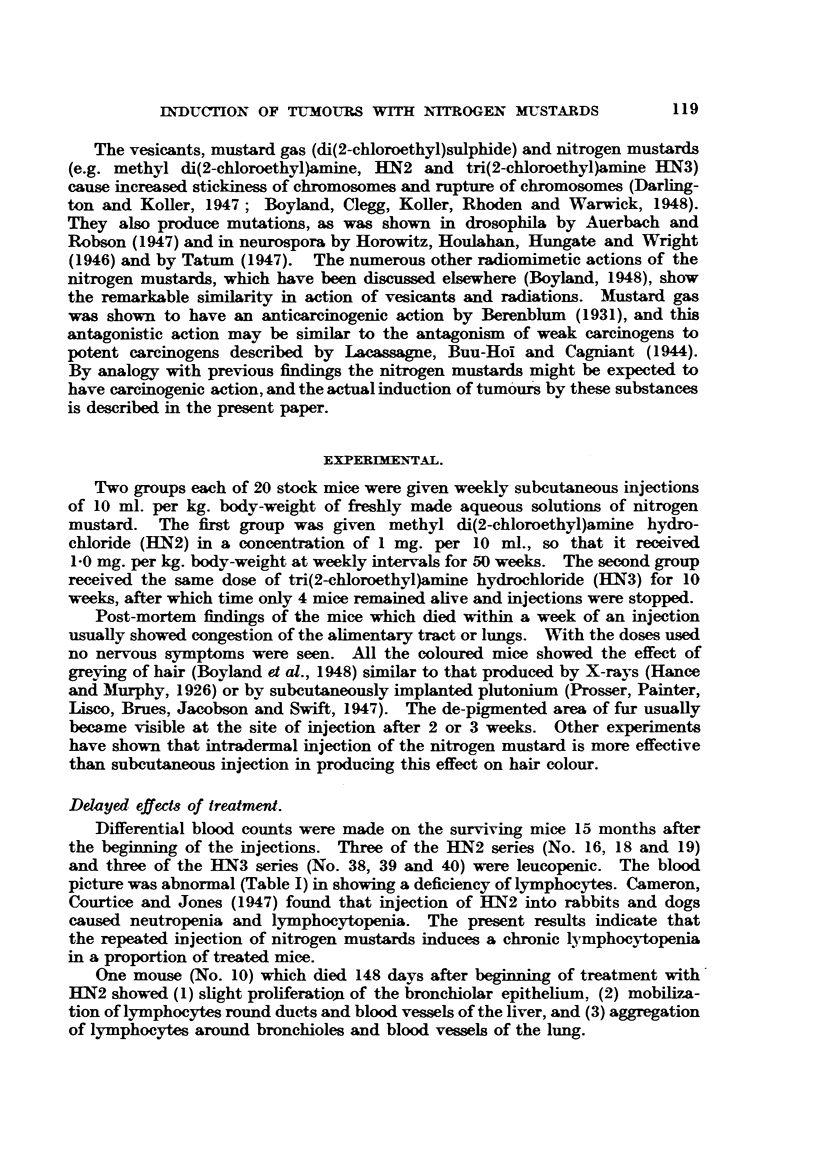

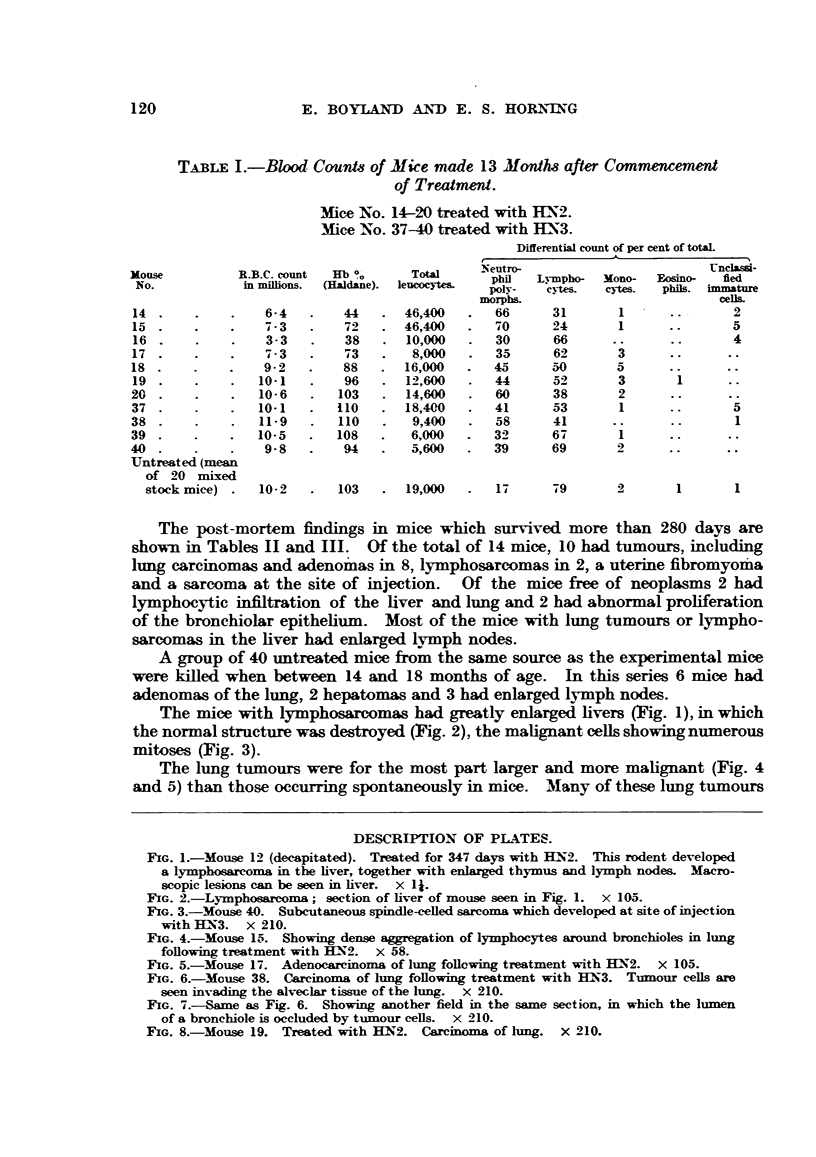

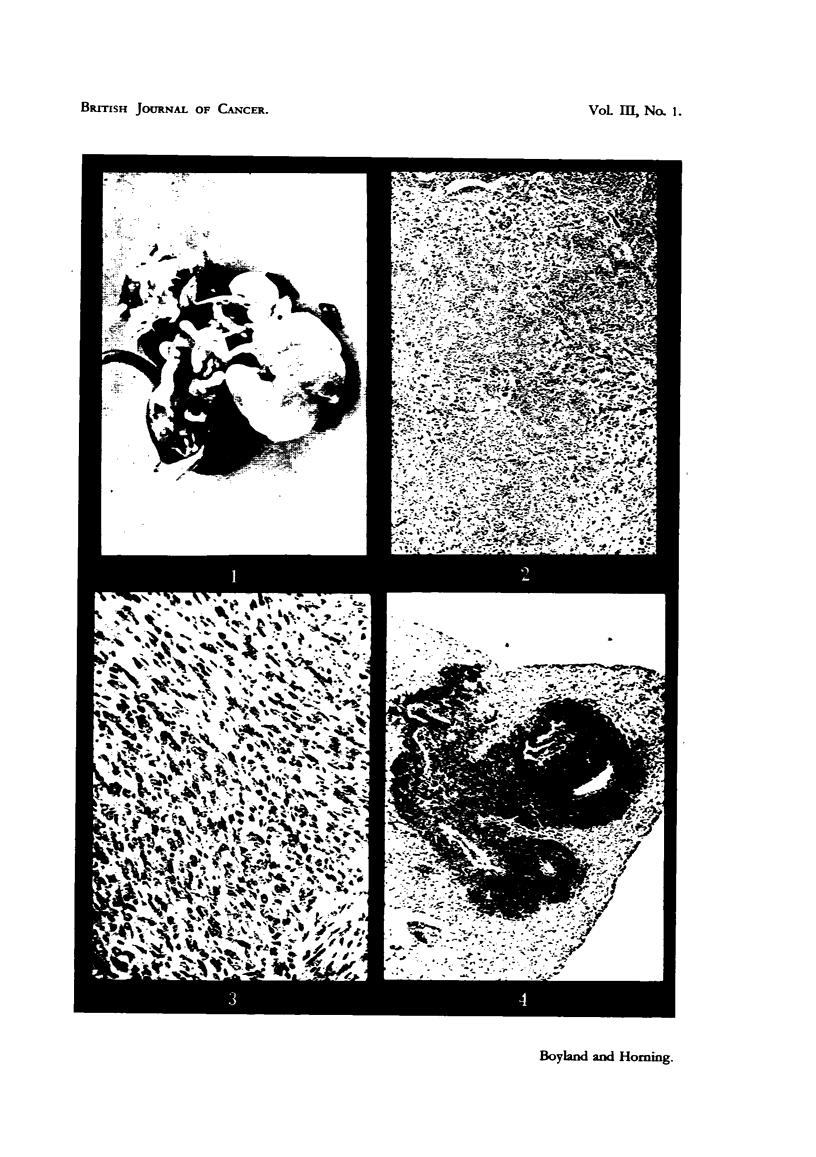

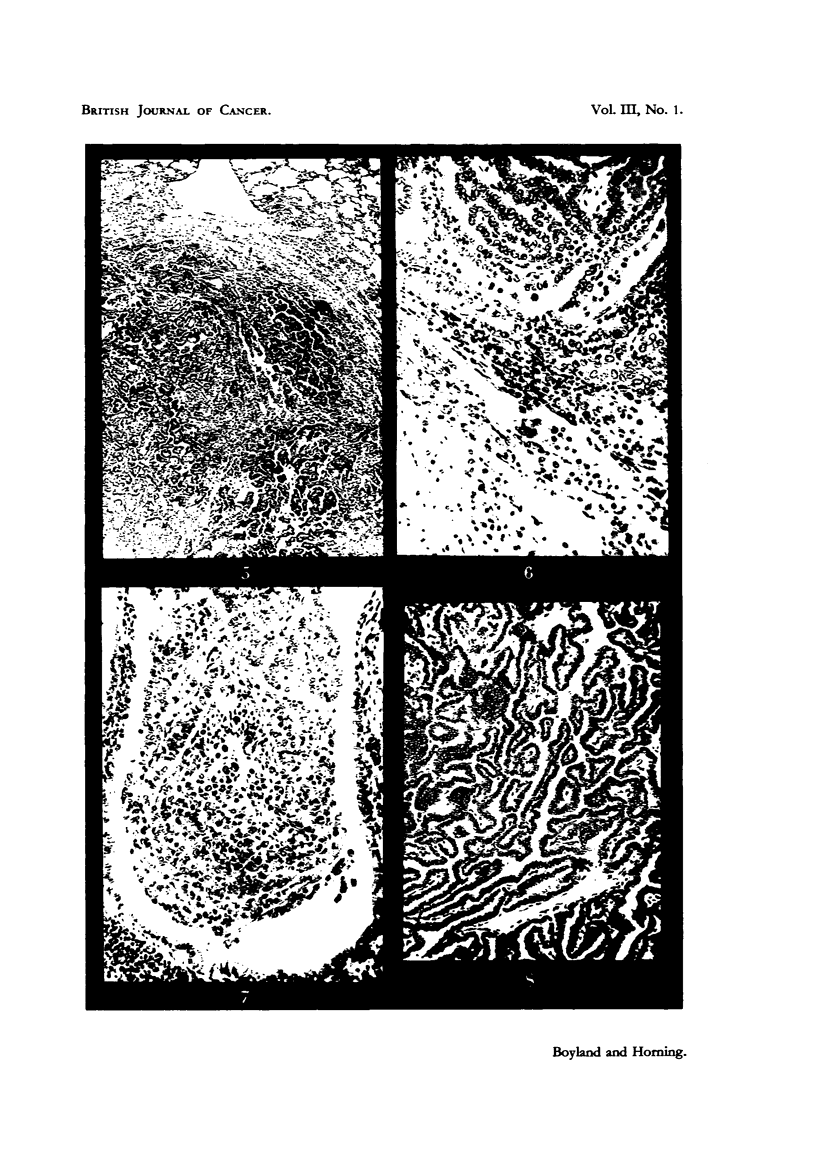

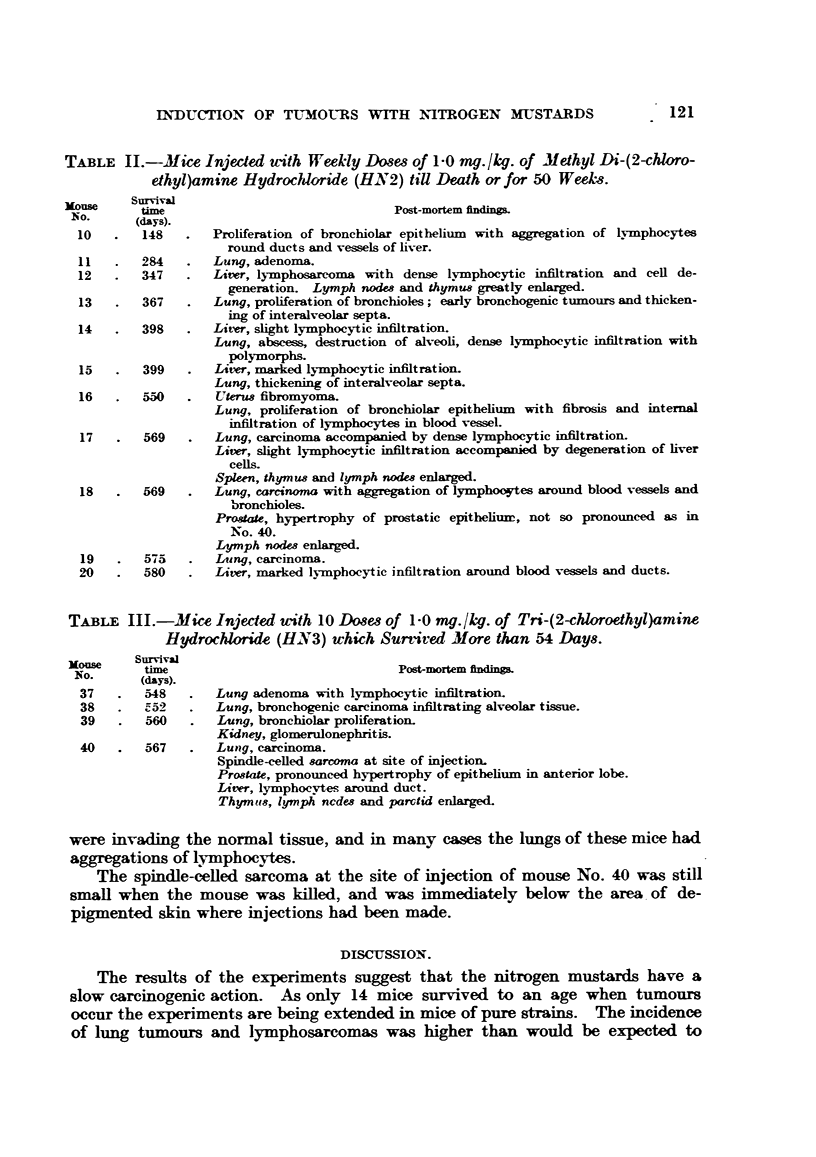

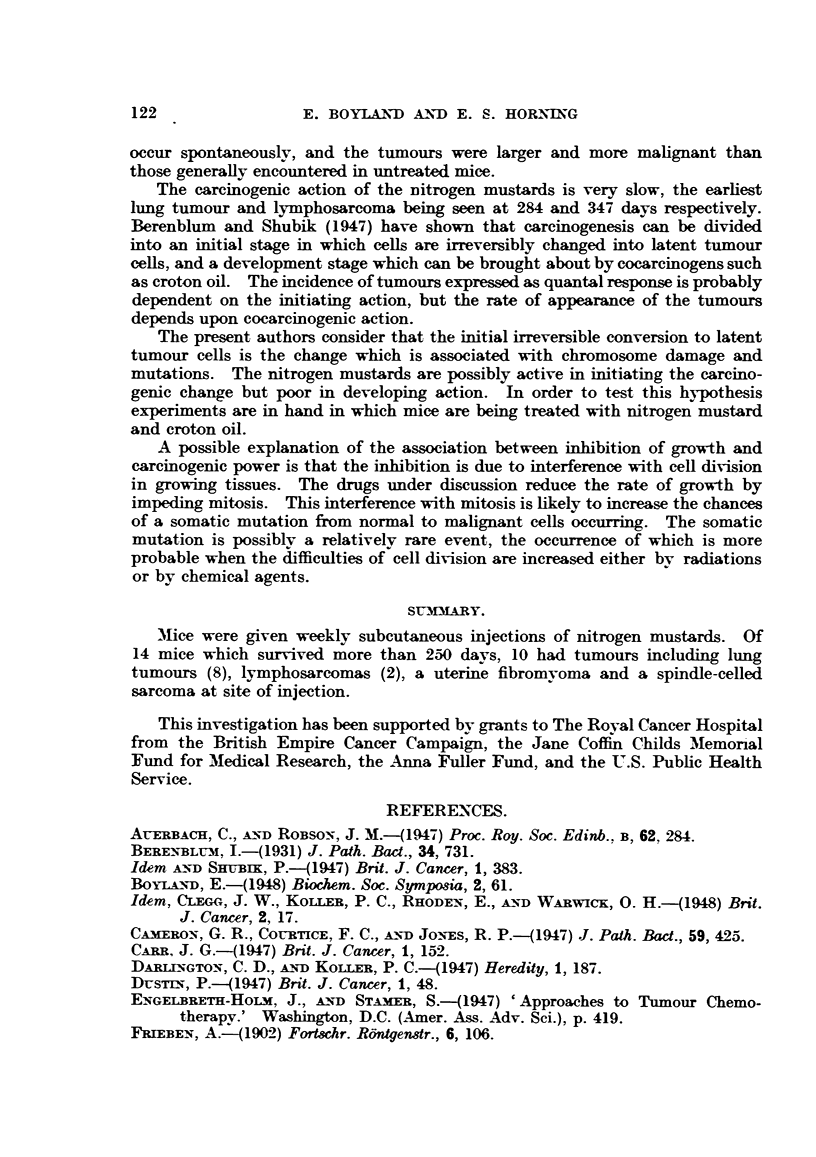

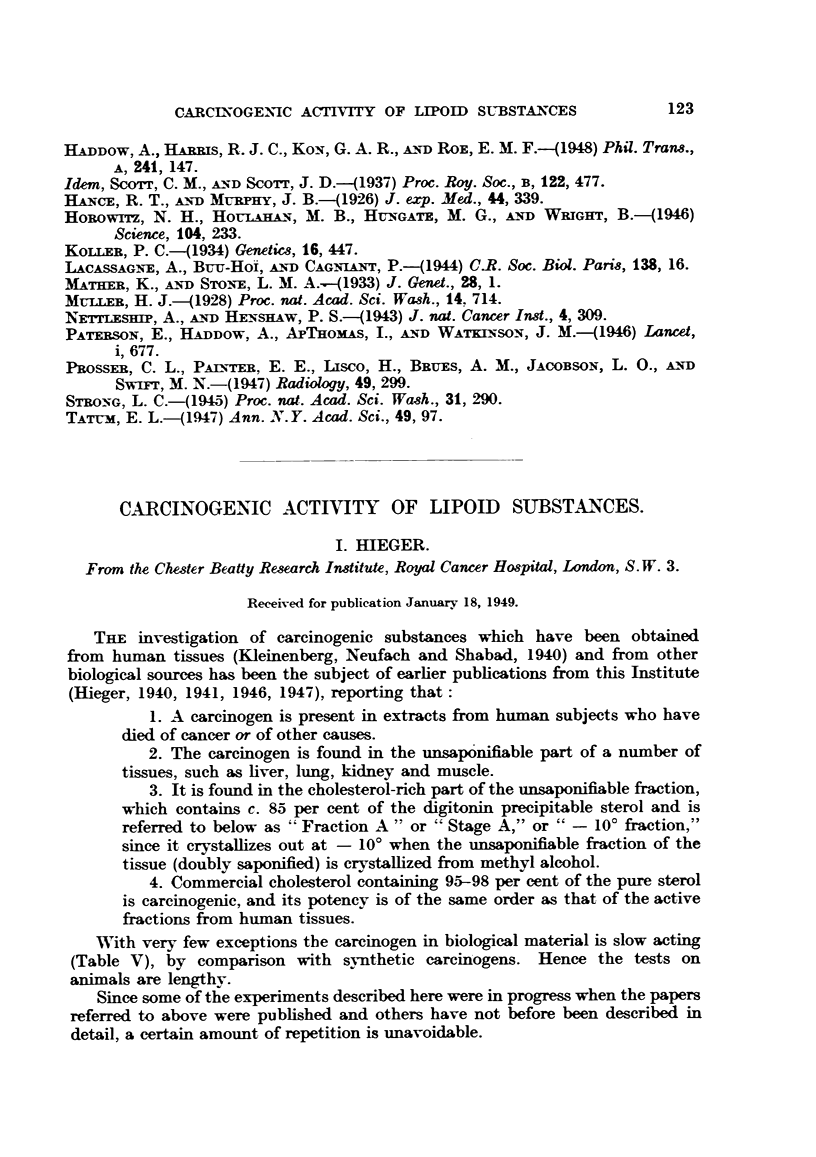


## References

[OCR_00438] Horowitz N. H., Houlahan M. B., Hungate M. G., Wright B. (1946). Mustard Gas Mutations in Neurospora.. Science.

[OCR_00458] Strong L. C. (1945). Genetic Analysis of the Induction of Tumors by Methylcholanthrene: XI. Germinal Mutations and Other Sudden Biological Changes Following the Subcutaneous Injection of Methylcholanthrene.. Proc Natl Acad Sci U S A.

